# Case of Temporary Loss of Vision, Depending upon Morbid Excitability of the Spinal Motor Nerves

**Published:** 1851-06

**Authors:** 


*Case of temporary loss of Vision, from dilatation of the Pupils, which frequently occurred, and depended upon morbid excitability of the Spinal Motor Nerves of the radiating fibres of the Iris,— offered as evidence of the existence of such Nerves, and because of its practical importance.* By C. H. Stone, M. D., of Natchez, Miss.

Mrs. A. B., (the initials are fictitious,) aged about twenty-four years, had always enjoyed good health, interrupted only slightly for a year before marriage, when she became affected with a morbid sensibility of the sacral nerves, and from which, probably, resulted an abortion in June, 1846, attended with considerable hemorrhage, which continued moderately, with occasional cessation, for a month. Her general health was restored by a visit to the seashore; the undue sensibility of the sacral nerves was overcome by cupping and counter irritation. She became pregnant, and was delivered, at full term, in July, 1847.

While much debilitated and unable to sit up, in consequence of the abortion in 1846, she occupied her time, for nearly a month, almost continually, in reading and fine sewing. From that period, she dates an impairment of vision — an amaurotic state of both eyes, which was shown by variously colored objects appearing to float before the eyes, and by a dimness and momentary loss of vision suddenly taking place. This condition of her sight being only occasional, and not of much extent, except for two months previous to her confinement in 1847, was not communicated to her medical attendant until March, 1848. The floating of objects before the eyes, instead of increasing, as in amaurotic conditions, gradually and entirely disappeared soon after the confinement, but the sudden blindness became more and more readily and frequently produced.

Vision is (March, 1848,) perfect for a time, even to distinguish minute objects at a distance. The pupils contract and expand properly on exposure to, and removal from ordinary light, and as long as they remain of their usual size, the sight is perfect; but a dilatation suddenly takes place from exposure to a strong light, from reading or sewing, and even from looking intently upon an object for a short time, and instantly there is total blindness. Vision is again soon restored, even while the eyes remain fixed upon the object.

There is no uneasiness in the eyes or head, nor do the former appear in the least affected, except as stated.

The general health is much impaired; she has fever; the urinary secretion is very scanty and highly colored, and the ankles are swollen and very painful. A few moderate portions of mercurial and laxative medicine, and diuretics of acetate of potash and colchicum, gradually and fully restored the general health, but the impairment of vision remained the same. I was therefore led to investigate the case still further, and I found that pressure over the second cervical vertebra instantly caused dilatation of the pupils and total blindness. This was repeatedly tried at different times, with the same results, and every precaution taken to prevent deception, from its being produced by the other causes referred to, while this experiment was being made. If pressure was made often at the same visit, a longer time elapsed before vision became restored than when the neck had not been irritated by these means, and to such an extent that the patient once requested me to desist. She became satisfied, as I did, that the pressure produced the blindness, and recollected that she had previously observed the same effect from accidentally irritating that part of the neck.

After two months' treatment, an improvement took place, but not to a sufficient extent to satisfy me, and I therefore advised her husband to take her to the north for the benefit of a change of climate, and of other medical advice. Her case was examined by two distinguished physicians of the city of New-York, Drs. Cheeseman and Wallace, one, or both of whom, at first, discredited the idea I had advanced respecting the immediate cause of the blindness, and its production by pressure on the neck, but became satisfied of its correctness. Their treatment did not materially differ from that I had adopted. She returned in the fall, and, by the end of the year, became nearly relieved, and has since only had slight returns, not sufficient to induce her to use any remedy. In the latter part of 1849, she was again delivered at full term, and remains in good general health.

Although I had not seen a similar case, I was not surprised at the development of what was evidently the source of the blindness. It was plain that the loss of vision depended solely upon the dilatation, for it was evident that the retina was in a normal state, because, during the blindness, the patient was fully conscious of light, and, with the exception stated, vision was as distinct and perfect as ever, for small as for large objects, and whether those were viewed at short or long distances.

The blindness seemed to result from a want of power to adjust the forces of the muscular tissue of the iris, or rather to *sustain* the adjustment, and this to depend upon a morbid excitability of the radiating fibres, which contracting suddenly and excessively, dilated the pupils. The power of regulating the admission of a due quantity of light was, in this way, lost — vision was overwhelmed by too much light.

It would have been more in accordance with present belief on this point, to have attributed the dilatation to a want of power in the motor nerves of the circular fibres, to maintain a due contraction of the pupils, which might result either from a deficiency of excitability in themselves, or of sensibility of the ophthalmic branch of the fifth, or from some impairment of the retina, but as an excitant to the cervical region caused the dilatation, such suppositions would, from this fact alone, have been highly unsatisfactory in this case, however well the fact of such impairments might explain a dilatation and blindness in other cases. It was an active, not a passive, dilatation. It yet remained to be explained how the dilatations resulted from an *excitation* made through the eye, a *contraction* of the pupil being its usual, and, indeed, only recognized effect.

It was reasonable to infer that the spinal motor nerves, if such existed, being in a state of morbid excitability, would receive an impression from an undue excitant in preference, or in *preponderating proportion*, to the motor nerves of the circular fibres, which were as reasonably supposed to be in a normal condition. That the reverse of this takes place in a physiological condition seemed not a valid objection to this explanation.

I was led to this examination and opinion of the case from what I had often seen in cases of irritation of spinal nerves — *motor* as well as *sensitive*, which it had been my fortune to witness, from the time the attention of the profession had been more fully called to the latter class of cases by Teale's work on Neuralgia, in 1829. Instances in which the sensitive nerves have been in a state of morbid sensibility, have, since then, been often observed and reported, while the morbid excitability of the motor nerves has been much overlooked. I had seen attacks of violent spasmodic cough, distressing dyspnœa, palpitation, vomiting, eructation of gas, &c., brought on instantly by pressure on the spinal column, thus developing the source of otherwise obscure and sometimes dangerous disorders. I therefore did not hesitate to form and express my opinion of this case to the patient and her husband, but I felt it my interest and duty to investigate it thoroughly, or to the extent of my ability; and I am free to admit that I was not so well acquainted with the minute anatomy and physiology of the eye, as to know whether the *authorities* would confirm my views, or render them wholly untenable, and leave my fact inexplicable.

It was no objection to my opinion, that Todd and Bowman, and other anatomists, as far as I could learn, did not describe *motor* nerves to the radiating fibres of the iris, for proof of such might be derived from experimental and pathological facts, more certainly than from anatomy, the more especially as the circular and radiating fibres are allowed equally to possess the character of muscular tissue; and as circular fibres are admitted to have a motor nerve for the contraction of the pupil, so the radiating fibres ought to have a motor nerve for their action and the consequent expansion of the pupil. The idea of a passive contraction of the latter, if tenable in any case, was not in this. I found in the British and Foreign Medical Review, for July, 1846, p. 284, that 'some experiments of Signor Guarini would make it probable, not only that the movements of the iris are mainly due to two sets of muscular fibres,— one circular, for the contraction of the pupil, the other radiating for its dilatation — but, also, that these movements are under the influence of two sets of nervous fibres, of which one set, distributed to the circular fibres, are derived from the third nerve, through the ophthalmic ganglion, and the other, distributed to the radiating fibres, are supplied by the branches of the cervical spinal nerves which pass through the superior cervical ganglion of the sympathetic.'

The case of Mrs. A. B., received a full explanation from the experiments of Guarini, and also presented a pathological proof of the truth of his deductions. At that time I had not access to the experiments of Valentin, which are alluded to in this notice of Guarini's, but I have since procured the 'Physiological, Pathological and Anatomical Researches of Dr. John Reid,' republished in 1848, in which (at pp. 303 and 304,) he refers to the results of Valentin's experiments, from which it will be seen that what was made probable by the experiments of Guarini, in 1844 and '45, was rendered *more* than probable by those of Valentin in 1829.

Dr. Reid states that —'From these and other facts, Valentin concludes that the iris derives its motor filaments from two sources — from cerebral and from spinal nerves; its cerebral motor filaments come from the inferior branch of the motor oculi nerve; its spinal motor filaments from the spinal cervical nerves:' and that 'the cerebral filaments move the circular muscular fibres of the iris, or the contractor muscle of the pupils; the spinal filaments move the radiating muscular fibres of the iris, or the dilator muscle of the pupil.' 'Valentin likewise believes that this view of the motor nerves of the iris, being derived from two different sources, and supplying antagonist muscles, will not only explain the effects of lesion of the sympathetic and vagus in the neck upon the iris, *but enable us to understand the variety in the condition of the pupil as to contraction and dilatation, in certain diseases, which has hitherto puzzled medical men;* and also clear up some anatomical anomalies in the origin of the ciliary nerves, which have been recorded.'

This was highly satisfactory; but I did not expect to find the record of any case similar to the one I had met with, yet, at length, I saw an essay on 'Irritation of Spinal Nerves,' by Dr. Brown, published in 1828, in the Glasgow Medical Journal, and soon after republished in this country in the Journal of Foreign Medicine, volume 2, in which (page 322,) Dr. Brown describes the case of A. S., a girl of seventeen years of age, in whom there was, in addition to the usual symptoms of this disease, palsy, nearly complete, of the left upper extremity. The arm was numb, and she could only move one of the fingers. She lost the power of swallowing any thing but liquids. These two symptoms remained, though almost every other yielded to the means employed. She had no pain in the head at this time, at least no fixed pain, nor any apparent affection of the mind. At one time the sight was dim and the pupils dilated; but I conceived that these, as in other cases, were owing to the spinal irritation of the nerves of the neck, not to any affection of the brain.' And at page 323, he says,—'It was formerly remarked, too, that Miss M. and A. R. were completely blind for some time. It appears, therefore, that this symptom is not unusual in cases of spinal irritation. I can not, however, explain, by any nervous communication, how this should happen. I can only say, that I have observed partial or total blindness in several cases in which the upper cervical nerves were in an irritated state, and from the frequency of its occurrence, I must suppose, that it was connected with this irritation, and arose from it.

'As this symptom occurred, in general, during my absence, I am not sure whether, in all the cases, the pupils were dilated or not, during the state of blindness. In A. R.'s case, and in that of A. S., they were dilated, and I presume that such was also the case in the others, as that was the character of the eye when I saw it next day.'

It is reasonable to suppose that pressure on the upper front of the cervical region would, in these cases, have caused the pupils to dilate, and there is no reason to doubt that the dilatation was the cause of the blindness. There is sufficient 'nervous communication' with the upper cervical portion of the spine, to explain 'how this should happen,' if the function be admitted, for anatomists describe that the superior cervical ganglion of the sympathetic is connected by large branches with the first, second and third cervical nerves, and that the carotid plexus, formed by an ascending branch from this ganglion, has various communications; among others, there is a branch to the ophthalmic ganglion, and one or two filaments to the third pair. This is as much as anatomy can do; experiments and pathology must explain the rest; and if pressure near the roots of these cervical nerves, one or other being in a state of morbid sensibility and excitability, produces dilatation of the pupils; and if, 'after the pupil of a live animal has contracted on irritating the third nerve, the superior cervical ganglion be irritated, '*the pupil dilates again*,' as asserted by Guarini and Valentin, it appears certain, and better attested by the pathological than the experimental fact, that filaments from the first, second, or third cervical nerves, are motors for the radiating fibres of the iris, which could be determined by irritating the branches before their connection with the ganglion. Although I am well aware that an irritant to a portion of the spinal column, the cord in a state of irritation, will sometimes develop *pain* in the nerves going *to* (why may it not cause *motion* through those proceeding *from?*) the cord at another and distant section, in the same morbid state, yet as, in the case of A. B., the irritant was over the second vertebra, it is a fair inference that the spinal motor of the iris is a filament of the second cervical nerve.

I have had proof in many instances by pain in the eye, from pressure on the neck, that a nerve of sensation also connects the eye and the spinal cord, and so no doubt have many others. If the iris has one kind of spinal nerve, it has the other; and if it be granted that irritating the cervical ganglion, or irritating the spinal column, when an irritation exists in the cord or at the roots of its nerves, produces an action of the motor nerves, this can only occur through a nerve of sensation, for a pure motor nerve does not act from the direct application of irritants, because not sensitive — it acts only through a nerve of sensation or volition. That it was through a spinal sensitive nerve that the motor of the radiating fibres of the iris was excited, in irritating the cervical ganglion, and from pressure on the second vertebra, in the case of Mrs. A. B., will be admitted by all whose minds are not pre-occupied with the doctrine of Bell — a host, it is true,— but likely soon to be few from an accumulation of opposing facts, and their exposition by Mr. Alexander Walker, to whom, it is hoped, justice will yet be done in the acknowledgment that to him, and not to Bell, we are indebted for the complete enunciation of the fact, that the anterior and posterior spinal nerves perform distinct functions, and of the *true* doctrine that the former, *not the posterior*, are sensitive, and the latter, *not the interior*, are the motor nerves.

Dilatation of the pupils may occur from opposite conditions of the cerebral and spinal motors of the iris, as well as from a blunted sensibility of the excitors of the former, from a lessened excitability of one set, the cerebral, or from an increased excitability of another, the spinal. Belladonna and strychnine, will each produce the same effect — the former by a sedative impression, direct or indirect, on the cerebral, and the latter by an exciting impression on the spinal motor nerves and column; and it is well to know that, in disease, dilatation and blindness may depend upon the same opposite states of the same distinct sets of nerves — of those belonging to the circular and those to the radiating fibres; and as section of the spinal motor, by removal of the cervical ganglion, will cause *contraction* of the pupil, it may be well to remember that the same may happen from a blunted excitability or paralysis of this nerve, however rare for this to occur from such cause,— probably not more so, or as difficult to distinguish, as a *dilatation* from the same state of the cerebral motors.

Case of spinal irritation are numerous in the South, and, although far more frequently met with among delicate women, are not as much confined to them as generally supposed. I have seen them, many times, among men, and more frequently with the black race, of either sex, than the white man; and while affections of the *sensitive* nerves are more frequent and prominent, it is not unusual to find the *motors* chiefly, and apparently solely, implicated. Nor is it always that these irritations, especially of the sensitive nerves, begin at the roots — they more often, indeed, commence in the expansions, in which they often persist, notwithstanding an appropriate treatment of the spine may have afforded much relief; and this is also sometimes the case in primary irritations of the roots, or of the columns of the cord at the point of entrance or exit of the nerves, if long neglected: not only the secretory and other functions become deranged, but I am fully satisfied that *organic* changes have sometimes resulted from irritations of spinal nerves, long neglected.—*New Orleans Medical and Surgical Journal.*

March 20, 1851.

